# Subcutaneous methadone is not different than transdermal fentanyl for postoperative analgesia in dogs with thoracolumbar disc disease, a prospective, randomised, blinded clinical study

**DOI:** 10.1186/s12917-025-04941-3

**Published:** 2025-10-03

**Authors:** A. F. Schütter, A. Verhoeven, J. Tünsmeier, S. B. R. Kästner

**Affiliations:** Small Animal Hospital, University of Veterinary Medicine Hanover, Bünteweg 9, 30559 Hanover, Germany

**Keywords:** Glasgow Composite Measure Pain Scale–Short Form (CMPS-SF), Colorado State University Canine Acute Pain Scale (CPS), Visual analogue scale, Pain, Spinal surgery, Methadone, Transdermal fentanyl

## Abstract

**Background:**

Thoracolumbar disc disease is a common neurological condition in dogs, which incorporates different pain components. Multimodal analgesic treatments, especially postoperatively, are often based on opioids and require an intravenous catheter for drug application. This might impede early physiotherapy and mobilisation. Different composite pain scales and sensory testing devices exist to evaluate postoperative pain behaviour in dogs. At present, no data are available to clearly recommend one tool or technique after spinal surgery over others. Therefore, the primary aim of this study was to evaluate whether subcutaneously applied methadone or transdermal fentanyl can offer sufficient postoperative analgesia in dogs after thoracolumbar neurosurgery without the need to maintain intravenous access. A secondary aim was to evaluate which type of pain recognition tool would be suitable for dogs in this clinical setting. The hypothesis is that both subcutaneously applied methadone and transdermal fentanyl solution provide adequate pain relief in dogs after thoracolumbar spinal surgery.

**Methods:**

In a prospective, randomised, blinded clinical study, fifty client-owned dogs were repeatedly evaluated for 96 h after undergoing spinal surgery. Treatment group M received 0.4 mg/kg methadone subcutaneously two hours before the start of surgery and every 6 h thereafter. Treatment group F received a topical application of 2.6 mg/kg transdermal fentanyl two hours before the start of surgery. Dogs were assessed via the Glasgow Composite Measure Pain Scale–Short Form (CMPS-SF), the Colorado State University Canine Acute Pain Scale (CPS), a Visual Analogue Scale (VAS) and von Frey Filaments. The treatment groups were compared via the Wilcoxon rank sum test. Correlations between the three pain scores were evaluated via Spearman’s rank correlation coefficient.

**Results:**

Over the whole study course none of the pain evaluation methods could demonstrate a significant difference in analgesic requirements between groups M and F (*p* < 0.05). In both treatment groups, the pain scores on all three scales decreased over time. The results of the different pain scales correlated moderately to strongly. Skin sensitivity assessed using von Frey filaments showed considerable individual variation among dogs, with most responding only to thicker filaments.

**Conclusion:**

Subcutaneous methadone or transdermal fentanyl can provide adequate postoperative analgesia in dogs after spinal surgery without an intravenous catheter. The CMPS-SF and the CPS could reliably be used in this category of animals.

## Background

Thoracolumbar disc disease is a common neurological condition in dogs [1]. The postoperative analgesic treatment of these patients might be challenging, as their pain can include neuropathic, inflammatory and nociceptive components [2]. Different strategies for analgesia after spinal surgery in dogs have been reported, which are often based on multimodal analgesic treatments, including local anaesthetic techniques and opioids [3–6]. Methadone might have advantages because of its antagonistic action at the NMDA receptors in addition to opioid receptor binding [7]. Dogs after spinal surgery might benefit from early physical rehabilitation [8]. Starting physiotherapy and hydrotherapy/water treadmill exercise from 24 h to 3 days post-surgery is proposed [8], which hinders or interferes with hygienic maintenance of an intravenous catheter. Furthermore, each venous catheter is associated with a risk for irritation of the vessel and unwanted effects such as thrombophlebitis. Therefore, establishing an opioid-based analgesic protocol without the need to maintain an intravenous catheter could be beneficial. Unfortunately, methadone has very low oral bioavailability in dogs, necessitating parenteral drug application. Dosing regimens using intravenous methadone are documented, but owing to the half-life of 3.9 ± 1.0 h, dosing every 4–6 h is needed [9]. Due to absorption rate-limited elimination [9] a possibility to overcome some of these challenges could be subcutaneous (SC) application of methadone. Half-life for 0.4 or 0.5 mg/kg SC methadone are documented to be 10.7 ± 4.3 h [9] or 3.8 to 5.4 h [10]. Plasma methadone levels 6 h after 0.5 mg/kg SC administration in dogs ranged from 18 to 28 ng/ml. Which is slightly above 17 ng/ ml, the plasma concentration known to cause thermal and mechanical antinociception in beagle dogs [11]. So likely SC methadone administration every 6 or even 12 h could be adequate [9], and no intravenous catheter would be needed. At present, one study in which 0.25 mg/kg or 0.5 mg/kg SC methadone was administered in premedication and 4 h later for tibia plateau levelling osteotomy in dogs demonstrated analgesia lasting up to 12 h after the first drug application in both treatment groups [12].

Another option to provide postoperative opioid analgesia to dogs without the need to maintain an intravenous catheter is the transdermal route. A spot-on fentanyl solution for dogs has been available for some time and had been proven to be effective following a variety of soft tissue and orthopaedic surgeries [13].

Several composite pain scales exist for evaluating postoperative pain behaviour in dogs [14–16]. Sensory alterations can be quantified via methods such as skin sensitivity testing or assessment of mechanical or thermal thresholds, which appear to be more commonly used in research settings than in routine clinical practice [17, 18]. However, as pain scales focus on the assessment of behavioural based end points and the emotional part of pain, whereas stimulation tests rather evaluate the sensory part of the pain experience, a combination of techniques might be favourable in dogs with neurological disease. At present, no data are available to clearly recommend one pain type or sensation recognition tool or technique after spinal surgery over others.

Therefore, the primary aim of this study was to evaluate whether subcutaneously applied methadone or transdermal fentanyl can offer sufficient postoperative analgesia in dogs after thoracolumbar neurosurgery without the need to maintain intravenous access. A secondary aim was to evaluate which type of pain recognition tool/technique or combination would be suitable in dogs with thoracolumbar disc disease in a clinical setting, as at present, no specific pain scale for evaluating dogs after neurosurgery is available.

The hypothesis is that both subcutaneously applied methadone and transdermal fentanyl solution provide adequate pain relief in dogs after thoracolumbar spinal surgery.

## Methods

The study design was ethically reviewed and approved by the Lower Saxony State Office for Consumer Protection and Food Safety (LAVES), according to the regulations of the German Animal Welfare Act (AZ: 13A383).

The trial was designed as a prospective, randomised, clinical study with the investigator unaware of the opioid-analgesic treatment of the dogs during the entire study period. Randomisation was achieved via a commercially available website (www.randomization.com). Dogs were privately owned and regular patients of the hospital. Written informed consent was obtained from the owners prior to study enrolment. The inclusion criteria were as follows: age > 12 months and thoracolumbar intervertebral disc extrusion requiring hemilaminectomy. The exclusion criteria were as follows: age < 12 months, highly aggressive demeanour, need for revision surgery during the first 4 days after initial surgery, and known or suspected intolerance to one of the drugs used in the trial.

Treatment group M (M) received 0.4 mg/kg methadone (Comfortan, CP-Pharma, Burgdorf, Germany) applied subcutaneously (SC) in the region of the ventrolateral thoracic wall two hours before the start of surgery and every 6 h thereafter. Treatment group F (F) received a topical application of 2.6 mg/kg liquid transdermal fentanyl (Recuvyra 50 mg/ml, Eli Lilly & Co., Ltd., Elanco Animal Health, Hampshire, UK) two hours before the start of surgery. Following the manufacturer’s recommendation, fentanyl was applied to the neck region by a veterinary professional wearing personal protective clothing consisting of gloves, a gown and eye protection. After fentanyl application, dogs were gently restricted from movement for two minutes to prevent shaking and inadvertent spread of the drug.

Intravenous (IV) anaesthesia premedication consisted of 0.4 mg/kg levomethadone in a fixed combination with fenpipramid (L-Polamivet, Intervet Deutschland GmbH, Unterschleißheim, Germany) and 0.5 mg/kg diazepam (Diazepam ratiopharm, Ratiopharm, Ulm, Germany). Propofol (Narcofol, CP-Pharma, Burgdorf, Germany) IV dosed to effect was used to induce anaesthesia. After endotracheal intubation using an endotracheal tube with a cuff, anaesthesia was maintained using isoflurane (Isofluran CP, CP-Phama, Burdorf, Germany) in oxygen, which was delivered via a circle breathing system. Electrocardiogram, oscillometric blood pressure, percentage of oxygenated haemoglobin, pulse rate, respiratory rate, expiratory carbon dioxide and isoflurane, as well as body temperature were monitored using a multiparameter anaesthesia monitor.

Postoperative analgesia included the study drug (methadone or fentanyl) and additionally 4 mg/kg pregabalin (Lyrica, Pfizer Manufacturing Deutschland GmbH, Freiburg, Germany), which was given orally every 8 h. Based on pain scores and the appearance of dysphoria rescue analgesia consisting of 0.2 mg/kg methadone IV in dogs judged to be not dysphoric (Comfortan, CP-Pharma, Burgdorf, Germany) or 50 mg/kg metamizol IV in dogs showing dysphoria (Novacen, CP- Pharma GmbH, Burgdorf, Germany).

All non-analgesic medical treatment was at the decision of one of two residents of the European College of Veterinary Neurology (ECVN) under supervision of the local ECVN diplomate, who oversaw the postoperative care of the dogs. Both were not involved in this trial but were aware of the aim and course of the study.

Over the course of the study, each dog was evaluated by the same researcher.

Patient assessment always started with the determination of a sedation score, which ranged from zero to 3. Zero indicating a completely awake dog, one indicating a dog that appeared slightly sedated when undisturbed, but reacted to verbal approach, two indicating a dog that appeared sedated but reacted to light touch, three indicating a dog that appeared sedated but was rousable due to loud noise or firm tough or a dog which was not rousable at all. The first evaluation point was two hours after extubation. If the sedation score was ≤ 2, pain evaluation was initiated. If the dog was still sedated (score > 2), pain evaluation was started two hours later. After the initial pain assessment, measurements for the trial were repeated every 12 h over the next four days. In case rescue analgesia was needed, the dogs were evaluated 30 and 60 min after rescue drug application.

Skin sensitivity was tested via a set of classical von Frey Filaments in ascending order. The filaments were placed perpendicular to the skin, and the force was increased until either the dog showed a purposeful reaction (looking towards the filament, withdrawing from the filament), a panniculus reflex or the filament was maximally bent. If a reaction was observed, the weight of the filament was noted, and the von Frey Filament examination ended at this point. If no reaction of the dog was visible, the next larger filament was tested. A minimum time of 10 s was allowed between testing different filaments in one location. The measurement locations were bilaterally 0.5 cm, 5 cm and 10 cm lateral to the surgical wound at half the length of the wound.

The pain evaluation included the Glasgow Composite Pain Scale – Short Form (CMPS-SF), the Canine Acute Pain Scale (Colorado State University [CPS]) and a Visual Analogue Scale (VAS). All three pain scores were recorded via paper forms. For each individual dog, the scores were completed in the same order. However, the order of the three pain scales was randomised among the animals. Section B of the CMPS-SF was excluded from scoring in cases of non-ambulatory dogs.

### Statistical analysis

An a priori sample size calculation (G*Power 3.1.9.7; Heinrich Heine University, Germany) setting α = 5%, 1-β = 80%, and a difference of 1 for GCMPS between groups M and F indicates that 24 dogs would be necessary in the trial.

For statistical analysis, SAS (statistical analysis system) and GraphPad Prism 4 (GraphPad Software, CA, USA) were used.

The data distribution was tested via the Shapiro‒Wilk test. Comparisons between treatment groups were evaluated via the Wilcoxon rank sum test. Spearman’s rank correlation coefficient was used to measure the correlation between the three pain scores. The level of significance was set to α = 5%.

An outcome-based (rescue analgesia needed or not needed) comparison between the CMPS-SF and CPS was performed via McNemar’s test followed by evaluation of the PABAK (prevalence and bias adjusted kappa) [19].

## Results

The demographic data were not different between the treatment groups (Table 1).

**Table 1:** Demographic data of 50 dogs included in the present study

Treatment group	Methadone	Fentanyl
Number of dogs (n)	25	25
Female (intact/spayed)	10 (5/5)	10 (0/10)
Male (intact/castrated)	15 (10/5)	15 (14/1)
Weight (kg)	12.6 ± 10.6	12.6 ± 7.8
Age (years)	6.7 ± 3.2	5.8 ± 2.2
Dachshund	(11/25)	(10/25)
Mixed breed	(1/25)	(8/25)
Beagle	(1/25)	(2/25)
Jack Russel Terrier	(2/25)	(0/25)
Bolonka Zwetna	(1/25)	(0/25)
Dogue de Bordeaux	(1/25)	(0/25)
Border Collie	(1/25)	(0/25)
Chihuahua	(1/25)	(0/25)
Cocker Spaniel	(0/25)	(1/25)
Coton de Tulear	(0/25)	(1/25)
German Shepherd	(1/25)	(0/25)
English Bulldog	(0/25)	(1/25)
French Bulldog	(1/25)	(0/25)
Harzer Fuchs	(1/25)	(0/25)
Havaneser	(0/25)	(1/25)
Malinois	(0/25)	(1/25)
Malteser	(1/25)	(0/25)
Pekinese	(1/25)	(0/25)
Shih Tzu	(1/25)	(0/25)

Total anaesthesia and surgery time in group M were 214.2 ± 51.15 min and 133.4 ± 55.4 min. In group F total anaesthesia and surgery time were 215.8 ± 55.95 min and 132.4 ± 57.6 min.

At no time point could any of the methods demonstrate a significant difference in analgesic requirements between group M and group F. At 2 h, 4 dogs in F had a sedation score > 2, which resulted in the evaluation of these 4 dogs 2 h later at 4 h.

Rescue analgesia was required by 1/25 dogs in group M and 3/25 dogs in group F. Four additional dogs in group M were scored ≥ 5 using the CMPS-SF at one measurement point on the first study day each, but clearly exhibited dysphoric behaviour (e.g., howling) which could not be interrupted by talking to the animal or approaching it. Therefore, metamizole rescue analgesia was provided to these dogs, and the methadone dose was reduced. At the reassessment 30 min post drug application, no dog appeared painful.

**Fig. 1:**
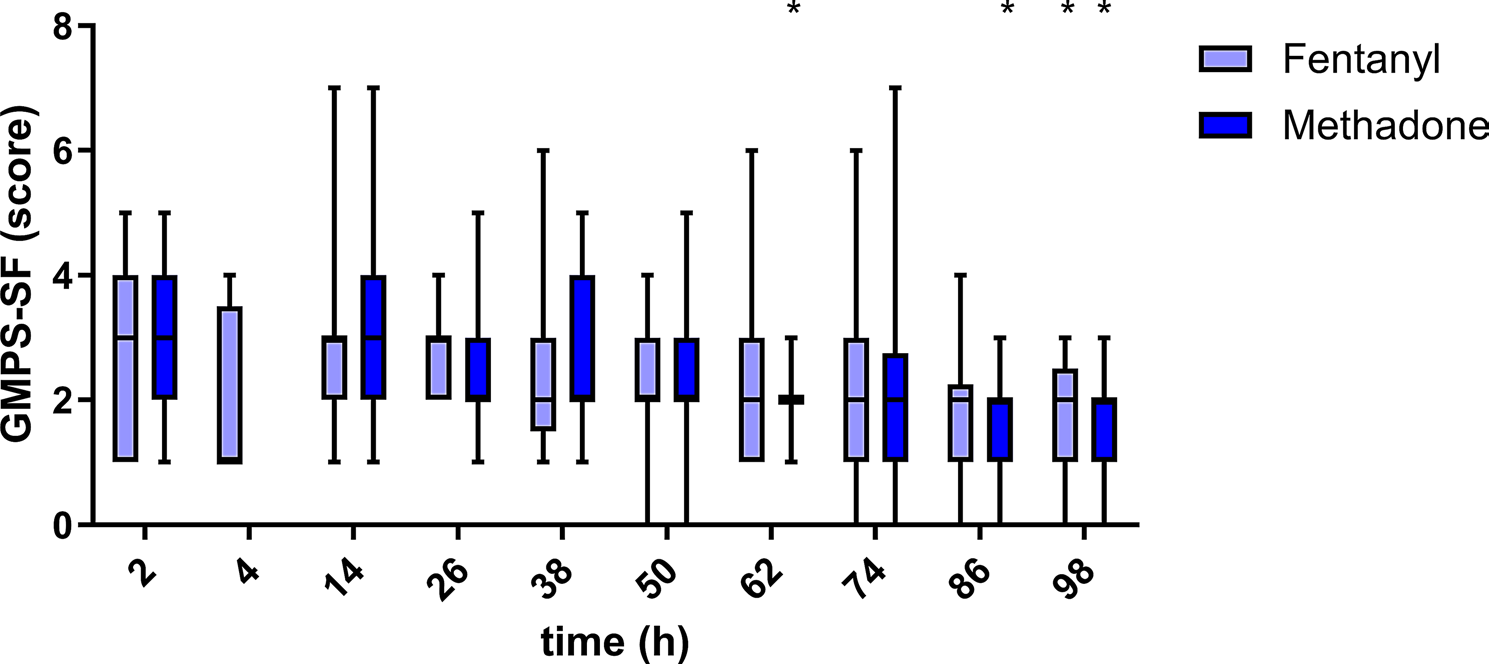
Glasgow Composite Measure Pain Scale–Short Form (CMPS-SF) over time for 25 dogs in group methadone and 25 dogs in group fentanyl. Boxes contain 50% of the data, the horizontal line within the box indicates the median, and the whiskers indicate the minimum and maximum values. Significant differences from the first measurement point (2–4 h post extubation) within each treatment group are indicated with an asterisk (*). Measurements at the 4 h time point were only performed if the dogs were too sedated at the 2 h time point to be evaluated. The CMPS-SF range was 0–20

**Fig. 2:**
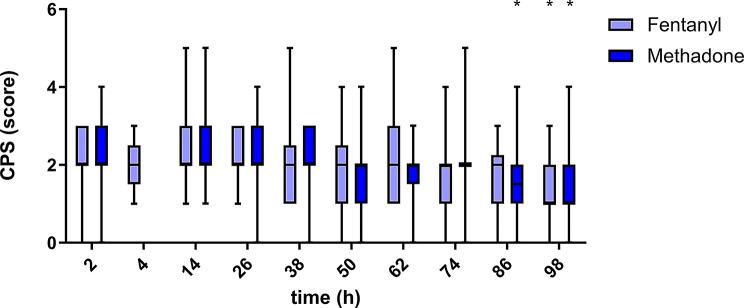
Colorado State University canine acute pain scale (CPS) over time for 25 dogs in group methadone and 25 dogs in group fentanyl. Boxes contain 50% of the data, the horizontal line within the box indicates the median, and the whiskers indicate the minimum and maximum values. Significant differences from the first measurement point (2–4 h post extubation) within each treatment group are indicated with an asterisk (*). Measurements at the 4 h time point were only performed if the dogs were too sedated at the 2 h time point to be evaluated. The CPS range was 0–12

**Fig. 3:**
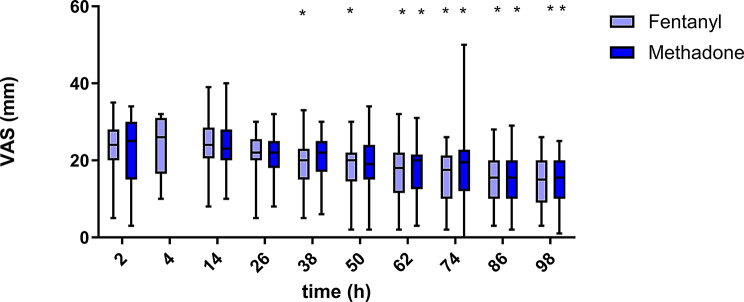
Visual analogue scale (VAS) over time for 25 dogs in group methadone and 25 dogs in group fentanyl. Boxes contain 50% of the data, the horizontal line within the box indicates the median, and the whiskers indicate the minimum and maximum values. Significant differences from the first measurement point (2–4 h post extubation) within each treatment group are indicated with an asterisk (*). Measurements at the 4 h time point were only performed if the dogs were too sedated at the 2 h time point to be evaluated. The VAS score ranged from 0–100 mm

In both treatment groups, the pain scores on all three scales decreased over time (Figs. 1, 2 and 3). The results of the different pain scales correlated moderately to strongly (Table 2).

**Table 2:** Spearman correlation between three pain scales, the visual analogue scale (VAS), the Glasgow composite measure pain Scale–Short form (CMPS-SF) and the Colorado state university pain scale (CPS), for treatment groups methadone and fentanyl. Values between 0.5 and 0.75 indicate a moderate correlation; values above 0.75 indicate a strong correlation

	Methadone	Fentanyl	*p*
CMPS-SF vs. CPS	0.70	0.73	< 0.001
CMPS-SF vs. VAS	0.60	0.64	< 0.001
CPS vs. VAS	0.62	0.70	< 0.001

Using the outcome-based measure “Is further analgesic treatment needed or not?”, in both treatment groups, very strong agreement between the CMPS-SF and CPS was demonstrated. For groups M and F, the prevalence and bias adjusted kappa coefficients were 0.86 and 0.93, respectively. Both scales lead to the same therapeutic decision in 215/231 (M) or 207/216 (F) measurements. The detailed results for the outcome-based measure are shown in Table 3.

**Table 3:** Outcome-based measures for the Glasgow composite measure pain Scale–Short form (CMPS-SF) and the Colorado state university pain scale (CPS) for treatment groups Fentanyl and methadone. A score of ≥ 5 or ≥ 4 indicates a need for further analgesia, and a score of < 5 or < 4 indicates an adequate pain state, according to the respective pain scale used

		Methadone		Fentanyl	
		CMPS-SF			
		< 5	≥ 5	< 5	≥ 5
CPS	< 4	208	5	203	1
	≥ 4	11	7	6	4

**Fig. 4:**
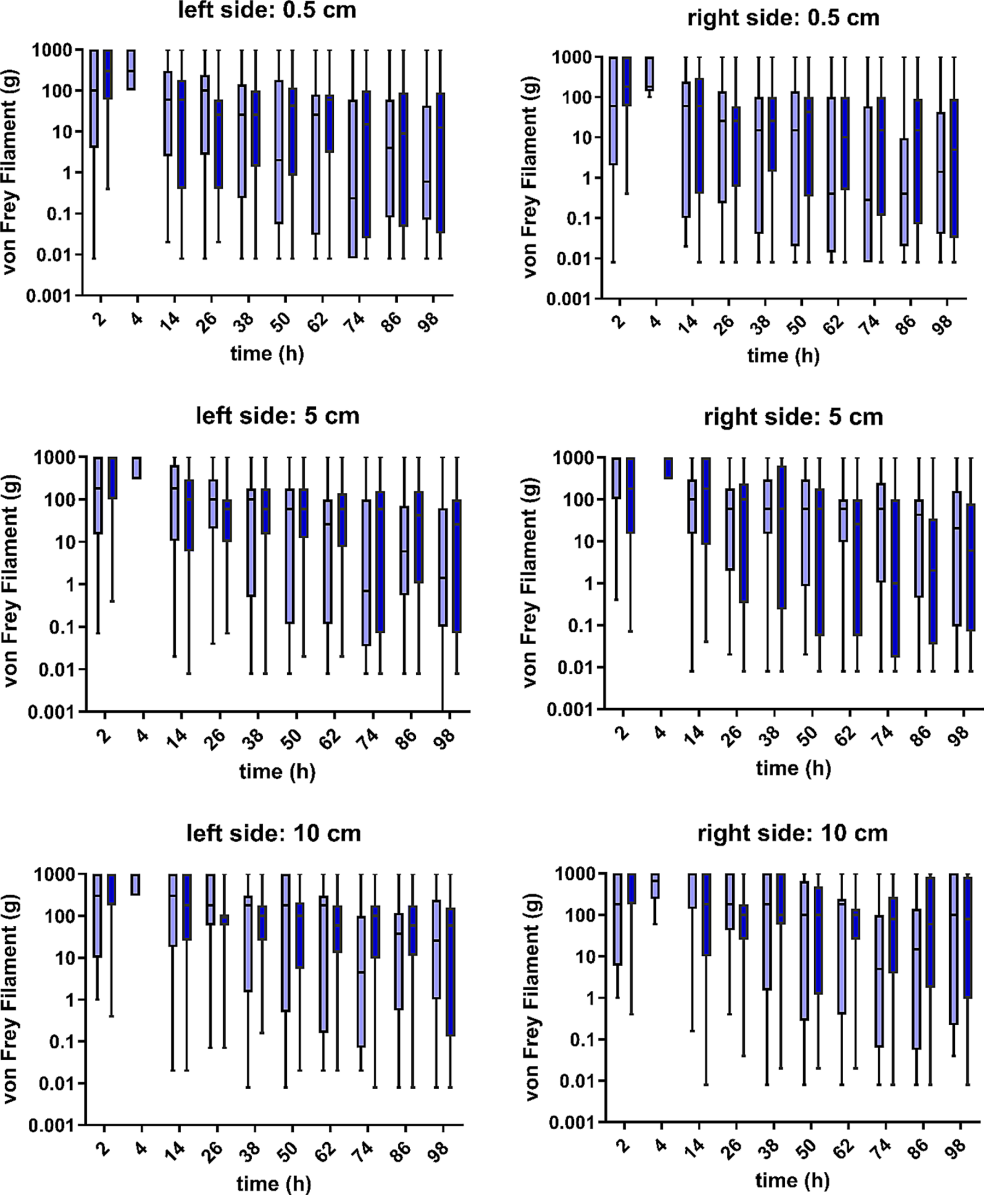
Von Frey Filaments were used over time for 25 dogs in group methadone (dark blue) and 25 dogs in group fentanyl (light blue). The reactions of the left and right sides of the body at 0.5, 5 and 10 cm distances to the surgical wound are shown. Boxes contain 50% of the data, the horizontal line within the box indicates the median, and the whiskers indicate the minimum and maximum values. For animals not reacting at all, 1000 g was set arbitrarily to display these dogs in the figure

When von Frey Filaments were used, no significant difference in skin sensitivity was detected between groups M and F. The results for skin sensitivity showed large individual variation, with a tendency toward reactions only in thicker filaments. Across all the measurements, in 15% of measurements no positive reaction was visible, and in another 26% of measurements filaments larger than 100 g were needed (Fig. 4).

Two dogs in group F were bradycardic (40–48 bpm) and hypothermic (34.8 °C) and received 0.01 mg/kg glycopyrrolium IM, active warming and intravenous fluid therapy using a balanced electrolyte maintenance solution.

Two dogs in group M showed mild to moderately increased salivation, two dogs in each treatment group demonstrated one episode of vomiting, and one dog in each treatment group developed mild diarrhoea; all gastrointestinal effects were self-limiting and did not require medical treatment.

All patients in this study received phenoxybenzamine and bethanechol. Further drugs used in individual dogs were cefuroxime, amoxicillin/clavulanic acid, omeprazole, calendula ointment, dexpanthenol ointment, and vitamin A-containing eye ointment.

## Discussion

The hypothesis that both subcutaneously applied methadone and transdermal fentanyl solution can provide adequate postoperative analgesia in dogs after thoracolumbar neurosurgery could be confirmed. All three used pain scales provided reasonable monitoring of the animals. In contrast, von Frey Filament testing did not appear helpful for the clinical evaluation of pain in these dogs after hemilaminectomy.

Owing to the low number of animals requiring rescue analgesia or showing undesirable effects, both analgesic courses (SC methadone and transdermal fentanyl) appear clinically comparable and adequate. Both drugs are µ-, κ- and δ- opioid receptor agonists [20, 21]. They are therefore classified as effective analgesics, which explains their effectiveness. However, based on in vitro studies, for both methadone isomers dose-dependent binding and antagonism at the NMDA receptor are being discussed [22, 23], which theoretically could be beneficial in dogs with spinal disease. Intervertebral disc disease often involves neuropathic pain components, which could benefit from drugs that influence NMDA receptor activity [24, 25]. An antihyperalgesic effect of the D- isomer of methadone has been documented in laboratory rodents [26]. However, in the studied clinical cases, no benefit of the possible NMDA action was detectable. Independent of the treatment group all dogs in the current trail received the same dose of levomethadone as part of anaesthesia premedication. It is possible that this still could have an analgesic effect at the first post operative measurement points and therefore could have blunted existing group differences during the early postoperative time. As no plasma levels of methadone and metabolites were measured this effect cannot be excluded.

Furthermore, the application of pregabalin to all dogs could have attenuated smaller differences between treatment groups M and F. Including pregabalin in the postoperative analgesic plan for dogs with disc herniation led to favourable analgesia, as shown by reduced pain scores compared with methadone alone [18]. However, only trends toward lower pain scores are documented when gabapentin is used in a similar setting [27]. Additionally, in humans, pregabalin is known to be effective in patients suffering from various diseases leading to neuropathic pain [28, 29]. Furthermore, opioid-sparing effects of pregabalin have been documented in humans and laboratory rodents [30, 31]. At present, no data concerning pregabalin opioid sparing in dogs exist. But plasma levels in dogs documented after 4 mg/kg orally applied pregabalin as a single dose (4.1 µg/ml) or after repeated dosing every 8 h (5.1 µg/ml) are in the range for which analgesia in humans is expected [18, 32].

The subcutaneous route for methadone application was chosen. At present, most postoperative analgesic plans, including methadone use intravenous drug administration [33, 34]. The literature concerning the subcutaneous use of methadone in dogs is limited [10, 12, 35]. The dosing regimens and durations of treatment used in these studies are diverse. Pharmacokinetic data in dogs, indicating a longer half-life of methadone when it was injected subcutaneously (10.7 ± 4.5 h) compared to when it was applied intravenously (3.9 ± 1.0 h) [9]. Further plasma concentrations 6 h after SC methadone application (18–28 ng/ml) are known to be just above the level causing thermal and mechanical antinociception in beagle dogs (17 ng/ml) [10]. On that basis a treatment of four times daily was scheduled [9, 10] for the current trial. Focussing solely on the documented mean half-life of methadone after subcutaneous injection, dosing only once or twice daily seems to be possible. However, owing to the rather large variability in half-life times, the data available about plasma levels and the lack of preexisting clinical data, more frequent dosing (four times daily) was performed. Judging the low need for rescue analgesia in the present study, this dosing regimen seems to be adequate for dogs after neurosurgery. However, four dogs in group M showed dysphoric behaviour, which resolved without medical intervention and did not reoccur after methadone dose reduction. This dysphoria could have been caused by high initial doses of methadone or accumulation due to frequent redosing. Therefore, based on individual patient evaluation reducing in methadone or extending application intervals could likely assist in prevention of dysphoria. Although not evaluated here, a reduced treatment frequency also might reduce stress and discomfort of patients due to reduced handling and might increase compliance with treatment in a busy clinical team. Furthermore, subcutaneous drug administration avoids the necessity of keeping a patent intravenous catheter and therefore can prevent possible painful incidents such as thrombophlebitis. This could also be avoided via the use of a transdermal fentanyl solution, which did prove to be effective and safe for the patients studied in the current trial. However, this specific fentanyl solution has been withdrawn from the market between the execution of the clinical trial and manuscript preparation and is currently (2025) not available. Fentanyl patches designed for application on human skin are still available, but owing to their different pharmacological properties, the results of this study cannot be transferred to the use of human fentanyl patches on dogs.

Methadone was administered four times daily over the course of the study, independent of the pain score of the animal. This alteration to clinical practice was taken to enable comparison to the long-acting transdermal fentanyl solution throughout the entire study. Therefore, the individual lowest effective dose was not titrated, and it might be, that some dogs in M received more methadone than they would have needed based on pain scoring. In a low number of dogs in group M mild unwanted gastrointestinal effects were present. It might be that they were caused by (to) high plasma levels of methadone. But the reason for these gastrointestinal effects cannot be determined with security. As vomiting and diarrhoea also occurred in single dogs in group F other factors like stress due to hospitalisation or changes in food compared to at home, could have caused them. The four-time daily subcutaneous methadone protocol seems to be suitable and safe for the majority of dogs after thoracolumbar spinal surgery. A dose reduction or extension of application intervals might be needed in individual dogs.

At present, no specific pain scale for evaluating dogs after neurosurgery is available; therefore, three different scales for the measurement of acute postoperative pain have been applied and compared. All three scales were able to detect postoperative pain behaviour, but the correlation between the two composite pain scales (CMPS-SF and CPS) was greater than that with the VAS. The VAS is considered a unidimensional scale that is based on the subjective impression of pain of the investigator [14, 15]. Hence, a very individual impression of the multimodal nature of pain is given. Both composite scales include three categories of evaluation: (a) observation of demeanour and posture, (b) approach to the animal and interaction, and C) touching the animal/painful area [36]. Following the steps of the scales, the observer is guided in how to evaluate possible pain; therefore, more aspects of pain behaviour are incorporated into the judgement in a structured way. This could explain why the composite scales correlate with each other more than with the VAS. Despite some differences in wording and the exact aspects that are evaluated, the CMPS-SF and CPS agreed concerning the outcome-based measure “Is rescue analgesia or more analgesia needed?” in 95% of all measurements. Considering the different intentions for which the scales were built, this might be of particular interest for institutions regularly working with untrained staff ([nurse] students, young veterinarians and nurses). The CMPS-FS was designed as a clinical decision-making tool, helping, together with the clinical judgement, to decide if an alteration in analgesic treatment is necessary [16]. In contrast, the CPS, which includes more visual aids, was created as a teaching tool, helping veterinary students identify behaviours, which could be caused by pain [37]. Keeping in mind that, in the present study, dogs were evaluated by a single person with moderate experience, both composite scales seem to reliably detect pain after neurosurgery and likely could be used interchangeably in these cases. The use of a scale that best fits the clinical situation (e.g., teaching hospital or not) therefore seems reasonable.

Von Frey Filaments were used to include a way of assessing dermal sensitivity and the presence of possible hypo- or hyperesthesia in the present study. The filaments at which the dogs responded were highly variable. Approximately 40% of the dogs responded only at one of the thickest filaments or not at all.

No distinct reason for this late reaction can be given. Dissection of nerves in the field of surgery could be one explanation, but using skin or skin and muscle incisions together with von Frey Filament application is an established model to evaluate postoperative analgesia, and a decrease in the reaction threshold is usually expected [38–40]. In addition, dermatomes often overlap; therefore, cutting the skin branch of a single spinal nerve should not result in increased thresholds [41]. Further, impairment of sensory neuron function on the level of the spinal cord, coursed by the disc disease could possibly also lead to reduced sensitivity. Despite the absence of statistical significance, a trend towards increasing sensitivity towards the end of the observation period was visible in the current study. The reason for this is not clear, but it might indicate the return of skin sensitivity. Furthermore, classical von Frey Filaments are used to detect changes such as hyperalgesia, which might develop after an insult [42]. Evaluating the dogs the first days after surgery might be too short for hyperalgesia or allodynia to be present. Further, the drugs applied could have influenced the results of von Frey Filament testing. Using an electronic von Frey device, an increase in thresholds after morphine application in dogs has been documented [43]. In addition to the µ-opioid effect, methadone acts antagonistically on spinal NMDA receptors, which could lead to an antihyperalgesic effect [7]. Pregabalin was applied to all animals in the present study. For pregabalin reducing cold and mechanical hyperalgesia in dogs with syringomyelia, chiari-like malformation or intervertebral disc disease has been demonstrated [17, 18]. Von Frey Filament testing was always the last step in evaluating a dog. Therefore, it cannot be excluded that some type of learning effect developed, leading to lower or later reactions of the dogs.

Nevertheless, the findings of the present study were similar to those reported in healthy dogs of ≤ 8 kg [44], which presented a response rate to von Frey Filament application in the thoracolumbar region of 56% and a tendency toward reactions with thicker filaments. Together with the fact that no clear hints for the need for rescue analgesia could be drawn from the data of the von Frey Filament tests, it is questionable whether the use of a test for skin hyperesthesia is a good choice for evaluating immediate postoperative analgesia. Perhaps measurements of classical mechanical thresholds via algometry would have been more useful.

The clinical nature of this trial causes some limitations of the study. The duration of disease before consultation in the clinic was not standardised; therefore, dogs with acute, subacute and chronic problems were included. This might have resulted in a heterogeneous study population but reflects the clinical situation. Pre-surgical pains scores as a baseline measure were not collected. They could be taken as an indicator of the severity of pain of the ongoing disease. But due to the clinical nature of the trial another impairment is that the dogs were enrolled independently of the analgesic treatment applied by the referring colleague. Therefore, it would have been questionable if a baseline pain score would really picture the baseline severity of pain or rather the efficiency of the already applied analgesic treatment. It remains unclear if and to what extent this medication could have influenced the results of postoperative patient evaluation. However, the drugs applied by the referring veterinarians most often were nonsteroidal anti-inflammatory drugs or steroids, which are likely not effective for longer than 24 h after application. Therefore, only the first measurement times might be influenced. Furthermore, including all possible cases reflects everyday practice and shows that the studied analgesic protocols appear to be effective and safe independent of previous medication. In addition, during the hospitalisation period, all medication, in addition to analgesic therapy, were at the discretion of the neurologist in charge and not standardised. All dogs included in this study received the parasympathomimetic bethanechol and the alpha sympatholytic phenoxybenzamine. For both drugs possible antinociceptive effects in laboratory rodents were discussed decades ago. However, whether these two drugs influenced pain modulation during the current trial cannot be proven. As no differences between the treatment groups and the applicability of the pain scores could be demonstrated, it appears highly unlikely that the additional medications have influenced the study results meaningfully.

All the animals were evaluated by only one investigator; to reduce stress to the hospitalised dogs, it was decided to refrain from examination by multiple assessors.

## Conclusion

Subcutaneous methadone or transdermal fentanyl can provide a similar degree of postoperative analgesia. This clinically appears to be an adequate postoperative analgesic treatment in dogs after surgery for thoracolumbar disc disease without the need to maintain an intravenous catheter. Both the CMPS-SF and the CPS could be reliably used in this category of animals after spinal surgery.

## Data Availability

Data is provided within the manuscript.

## Abbreviations

Cm: centimetre
°C: Degree Celsius
CMPS-SF Glasgow: Composite Measure Pain Scale -Short Form
CPS: Colorado State University pain scale
F: Fentanyl
Fig: Figure
H: Hour
IV: Intravenous
Kg: Kilogram
M: Methadone
Mg: Milligram
µg: Microgram
n: Number of dogs
NMDA: N-Methyl-D-Aspartat
SC: Subcutaneous
Tab: Table
VAS: Visual analogue scale
